# Plasma neurofilament light chain level predicts outcomes in stroke patients receiving endovascular thrombectomy

**DOI:** 10.1186/s12974-021-02254-4

**Published:** 2021-09-12

**Authors:** Chih-Hao Chen, Hai-Jui Chu, Yi-Ting Hwang, Yen-Heng Lin, Chung-Wei Lee, Sung-Chun Tang, Jiann-Shing Jeng

**Affiliations:** 1 Department of Neurology, National Taiwan University Hospital, Taipei, Taiwan; 2 Department of Neurology, En Chu Kong Hospital, New Taipei City, Taiwan; 3 Department of Statistics, National Taipei University, New Taipei City, Taiwan; 4 Department of Medical Imaging, National Taiwan University Hospital, Taipei, Taiwan

**Keywords:** Ischemic stroke, Biomarkers, Thrombectomy, Outcome, Hemorrhage

## Abstract

**Background:**

Timely endovascular thrombectomy (EVT) significantly improves outcomes in patients with acute ischemic stroke (AIS) with large vessel occlusion type. However, whether certain central nervous system-specific plasma biomarkers correlate with the outcomes is unknown. We evaluated the temporal changes and prognostic roles of the levels of these biomarkers in patients with AIS undergoing EVT.

**Methods:**

We enrolled 60 patients who received EVT for AIS and 14 controls. The levels of plasma biomarkers, namely neurofilament light chain (NfL), glial fibrillary astrocytic protein (GFAP), tau, and ubiquitin C-terminal hydrolase L1 (UCHL1), were measured with an ultrasensitive single molecule array before, immediately after, and 24 h after EVT (T1, T2, and T3, respectively). The outcomes of interest were death or disability at 90 days (defined as a modified Rankin Scale score of 3–6) and types of hemorrhagic transformation (hemorrhagic infarction or parenchymal hemorrhage).

**Results:**

Of the 180 blood samples from the 60 patients who received EVT, the plasma NfL, GFAP, and UCHL1 levels at T1 were significantly higher than those of the controls, and the levels of all four biomarkers were significantly higher at T3. Patients with parenchymal hemorrhage had a significantly higher rate of increase in GFAP (*P*_*interaction*_ = 0.005) and UCHL1 (*P*_*interaction*_ = 0.007) levels compared with those without parenchymal hemorrhage. In a multivariable analysis with adjustment for age, sex, National Institute of Health Stroke Scale score, history of atrial fibrillation, and recanalization status, higher NfL levels at T1 (odds ratio [OR] 2.05; 95% confidence interval [CI], 1.03–4.08), T2 (OR, 2.08; 95% CI, 1.05–4.01), and T3 (OR, 3.94; 95% CI, 1.44–10.79) were independent predictors of death or disability at 90 days.

**Conclusion:**

Among patients with AIS who received EVT, those with hemorrhagic transformation exhibited significant increase in plasma GFAP and UCHL1 levels over time. Higher plasma NfL were predictive of unfavorable functional outcomes.

**Supplementary Information:**

The online version contains supplementary material available at 10.1186/s12974-021-02254-4.

## Introduction

Acute ischemic stroke (AIS) with large vessel occlusion (LVO) has a considerably poorer prognosis than do other types of stroke [1]. When an intracranial artery becomes occluded, the brain tissues in the relevant region become ischemic within minutes and become irreversibly infarcted if the blood flow is lower than the threshold that brain tissues can tolerate. With advances in intravenous thrombolysis and endovascular thrombectomy (EVT), patients with AIS with LVO may benefit from timely recanalization to restore normal perfusion [2]. However, successful recanalization may not always translate into favorable outcomes because ischemia-reperfusion injury may result in progressive infarct growth or hemorrhagic transformation with detrimental effects; nevertheless, patients for whom recanalization is unsuccessful may experience persistent ischemia [3]. In such cases, the complex ischemic cascades, which involves interactions between infiltrated immune cells, circulating cytokines, activated platelets, the endothelium, and even central nervous system (CNS) cells, is a major contributor to post-stroke inflammatory responses that directly influence on the brain cell death [4].

Fluid biomarkers for such process involving ischemia-reperfusion and neuroinflammation may facilitate the prediction of stroke outcome. Studies have suggested that various circulating proteins are directly related to acute ischemic injury or inflammation of the CNS [5–7]. Brain tissues that become necrotic after stroke and the associated inflammation may release neuronal or glial-specific proteins into the cerebrospinal fluid and the blood, but their concentration in the peripheral blood is usually extremely low [8]. With advancements in detection technology, such as ultrasensitive immunoassays, these trace biomarkers can now be measured with confidence, potentially assisting in stroke prognosis prediction.

For example, a panel of single-molecule arrays focused on the CNS can simultaneously measure the levels of several biomarkers, including neurofilament light chain (NfL), glial fibrillary astrocytic protein (GFAP), tau, and ubiquitin C-terminal hydrolase L1 (UCHL1) [9, 10]. NfL is a neuronal scaffolding protein, and its levels in cerebrospinal fluid and plasma are elevated in individuals with neurological disorders, including acute stroke [11]. NfL may also reflect CNS inflammation-related injury and can be regarded as “the C-reactive protein of neurology” [12]. GFAP is a brain-specific intermediate filament protein that can distinguish intracerebral hemorrhage (ICH) from AIS according to the level of glial damage [13–15]. Tau protein is a critical biomarker of neurological disorders and is related to neuronal damage and blood–brain barrier instability [16]. UCHL1 is a neuronal cytoplasmic deubiquitinating enzyme with elevated levels in acute stroke [17].

Whether such a panel can be applied in patients with acute stroke treated with EVT remains unclear. We hypothesized that these plasma biomarkers would change with the recanalization status and progression of ischemia. Therefore, we evaluated the prognostic roles of these plasma biomarkers in patients with AIS with LVO receiving EVT, focusing on the changes in their levels before ET, immediately after EVT, and 24 h after stroke onset.

## Methods

The study protocol was approved by the Institutional Ethics Committee of National Taiwan University Hospital (NTUH-REC No. 201807029RINA). Written informed consent was obtained from all patients or their relatives. The study data are available from the corresponding author upon reasonable request by qualified investigators.

### Study population

Patients who experienced AIS and underwent EVT at National Taiwan University Hospital between February and December 2019 were enrolled. Participants without stroke from the cardiovascular department were recruited as the control group. The following clinicodemographic data were obtained from the patients with stroke: age; sex; medical history of hypertension, diabetes mellitus, hyperlipidemia, and atrial fibrillation; National Institute of Health Stroke Scale (NIHSS) score; causative occluded arteries; and use of intravenous recombinant tissue plasminogen activator. Causes of ischemic stroke were classified on the basis of the Trial of ORG 10172 in Acute Stroke Treatment classification as cardioembolism, large-artery atherosclerosis, or others [18]. The following clinicodemographic data were collected from the controls: age, sex, and medical history of hypertension, diabetes mellitus, hyperlipidemia, and atrial fibrillation.

### Endovascular treatment

The eligibility criteria for EVT were based on the guidelines published by the American Stroke Association [19]. In accordance with the protocol, after the stroke code had been initiated [20], the neurologist on duty visited the patient; computed tomography (CT) angiography with perfusion study was arranged if LVO was suspected [21]. Typically, in patients with anterior circulation stroke, EVT is indicated if the following criteria are met: (1) the patient has an Alberta Stroke Program Early CT Score (ASPECTS) ≥ 6, (2) occlusion is present in the internal carotid artery (ICA) or in segments M1 to M2 of the middle cerebral artery, and (3) groin puncture could be initiated within 6 h. For patients with (1) intracranial LVO of other arteries (e.g., the anterior cerebral, posterior cerebral, vertebral, or basilar arteries) or (2) an expected groin puncture time between 6 and 24 h, EVT was only indicated if a substantial mismatch between the infarct core and penumbra was observed through CT perfusion imaging.

The choice of the thrombectomy technique—stent retriever, thrombosuction, or a hybrid method—was made by the neurointerventionist [21]. Successful recanalization was defined as a grade of 2c or 3 on the modified Thrombolysis in Cerebral Infarction (mTICI) scoring system (grade 0, no perfusion; grade 1, minimal recanalization; grade 2a, partial antegrade reperfusion of <50% of the previously occluded target artery ischemic territory; grade 2b, antegrade reperfusion of ≥50% of the previously occluded target artery ischemic territory; grade 2c, near-complete reperfusion with slow flow or distal emboli in a few distal cortical vessels; and grade 3, complete reperfusion) [22]. The recanalization status and time metrics of the EVT procedure were recorded.

### Measurement of plasma biomarkers

All patients with stroke underwent peripheral blood draws at three time points: before groin puncture (T1), immediately after the completion of the EVT procedure (T2), and 24 h after stroke (T3). At each time point, 10 mL of venous or arterial blood was collected and centrifuged (2500*g* for 15 min) within 1 h of collection, and the plasma aliquots were stored in cryotubes at −80 °C until analysis. From the controls, 10-mL samples of venous blood were collected and prepared according to the same method. The plasma samples were tested through a Neurology 4-Plex assay established by the Simoa platform (Quanterix; Lexington, MA, USA); this assay can measure NfL, GFAP, tau, and UCHL1 levels simultaneously. The development of multiplex immunoassays for measuring CNS-related biomarkers would enable quicker and less expensive measurements through the use of samples with smaller volumes. The samples were analyzed by board-certified technicians blinded to the patients’ clinical status. All samples were analyzed in duplicates for inter-test validation, and the two results were averaged to determine the mean concentration. In addition, two internal quality control samples were required: one at the beginning and end of each run. The quality control samples for all four biomarkers were passed. For NfL, GFAP, tau, and UCHL1, the lower limits of detection were 0.104, 0.221, 0.024, and 1.74 pg/mL, respectively, and the average coefficients of variation were 4.8%, 4.6%, 10.5%, and 16.1%, respectively.

### Clinical and neuroimaging outcomes

To evaluate the clinical outcomes, all patients were followed up for 90 days after stroke. Their functional status was evaluated on the modified Rankin Scale (mRS), on which scores range from 0 (no symptoms) to 6 (death), with higher scores indicating more severe functional deficits. All patients were visited in person or contacted by phone for follow-up assessment, and the assessors were blinded to each patient’s biomarker status. To measure neuroimaging outcomes, data related to the initial CT perfusion study were collected and subjected to computer-assisted volumetric analysis (MIStar; Apollo Medical Imaging Technology, Melbourne, Australia) to quantify the infarct core and perfusion defect under a delay-corrected cerebral blood flow threshold of < 30% and delay time of > 3 s, respectively. The penumbra was defined as the difference between the infarct core and perfusion defect. Follow-up CT or magnetic resonance imaging was performed approximately 24 h after EVT to evaluate any hemorrhagic transformation and determine the final infarct size. Hemorrhagic transformation was further classified as hemorrhagic infarction (HI; Heidelberg Bleeding Classification of 1a and 1b) or parenchymal hemorrhage (PH; Heidelberg Bleeding Classification of 1c and 2) [23]. The lesions of the final infarct were judged on the basis of the corresponding hypodense areas on follow-up CT or hyperintense regions on diffusion-weighted magnetic resonance imaging, and OsiriX image analysis software (Pixmeo SALR, Geneva, Switzerland) was used to quantify the infarct volumes. The main clinical outcomes were death or disability (mRS score 3–6) at 90 days, whereas the main neuroimaging outcomes were the presence of any hemorrhagic transformation or PH.

### Statistical analyses

Because the levels of the four plasma biomarkers were not normally distributed, their values underwent natural log transformation before further analysis. The continuous variables are reported as a means ± standard deviations or medians (interquartile ranges), whereas the nominal and ordinal variables are presented as numbers and percentages. First, clinicodemographic data were compared between patients with favorable and unfavorable functional outcomes. The continuous and categorical variables underwent the Mann–Whitney *U* and chi-square tests, respectively. Biomarkers levels were compared between the controls and patients with stroke after adjustment for age because of the age imbalance. Next, linear mixed models were applied to evaluate changes in biomarkers levels across the three time points, and the data were stratified according to the patients’ status of recanalization (mTICI grade 2c or 3 vs TICI 0–2b), hemorrhagic transformation (no ICH, HI, or PH), and functional dependency status (mRS scores 3–6 vs. mRS scores 0–2) at 90 days. Spearman’s rank sum test was then used to determine correlations between plasma biomarkers and neuroimaging features, including ASPECTS, initial infarct core, initial perfusion defect, final infarct volume, and extent of infarct growth. To test for temporal relationship, estimations were made for the correlations of all neuroimaging features with biomarkers at T1 and with the final infarct volume and biomarkers at T2 and T3.

Furthermore, multivariable logistic regression models were applied to test the prognostic role of biomarkers in determining clinical outcomes, including death or disability at 90 days (mRS score: 3–6), any hemorrhagic transformation, and PH. Models were adjusted for age, sex, NIHSS score, and covariates associated with outcomes in the univariate analyses. These covariates included history of atrial fibrillation and successful recanalization for death or disability (Table 1) and hypertension for any hemorrhagic transformation (Supplemental Table I). No further adjustment was made for PH because of its rarity. The false discovery rate was used to control for potential errors during multiple comparisons of a given biomarker with outcomes at three time points. Finally, a generalized linear mixed model was employed to enable the repeated measurement of individual biomarkers at different time points, which served as independent variables, while the dependent variables and covariates were the same as  those used in the logistic regression models. All statistical analyses were performed using SAS software, Version 9.4 (SAS Institute Inc., Cary, NC, USA), and *P* < 0.05 indicated statistical significance.

**Table 1:** Comparison between favorable and unfavorable outcomes in patients receiving EVT

	All patients	mRS 0–2 (*n* = 32)	mRS 3–6 (*n* = 28)	*P*
Age	71.2±11.8	68.4±11.3	74.4±11.6	**0.04**
Male sex	34 (56.7%)	23 (71.9%)	11 (39.3%)	**0.01**
Hypertension	39 (65.0%)	19 (59.4%)	20 (71.4%)	0.33
Diabetes mellitus	22 (36.7%)	11 (34.4%)	11 (39.3%)	0.69
Hyperlipidemia	33 (55.0%)	17 (53.1%)	16 (57.1%)	0.76
Atrial fibrillation	37 (61.7%)	16 (50.0%)	21 (75.0%)	**0.046**
tPA administration	16 (26.7%)	7 (21.9%)	9 (32.1%)	0.37
NIHSS	16.3±6.9	15.1±6.3	17.7±7.5	0.18
Stroke subtype				0.59
Cardioembolism	43 (71.7%)	22 (68.8%)	21 (75.0%)	
Large-artery atherosclerosis	10 (16.7%)	5 (15.6%)	5 (17.9%)	
Others	7 (11.7%)	5 (15.6%)	2 (7.1%)	
ICA/M1 occlusion	44 (73.3%)	23 (71.9%)	21 (75.0%)	0.78
Procedure time				
Onset-to-puncture (min)	210 (138, 360)	239 (144, 418)	203 (134, 278)	0.30
Onset-to-recanalized (min)	234 (156, 391)	259 (159, 439)	215 (144, 336)	0.37
mTICI 2c-3	43 (71.7%)	27 (84.4%)	16 (57.1%)	**0.02**
Neuroimaging				
ASPECTS	8 (7, 9)	9 (7, 9)	8 (7, 10)	0.38
Initial core (ml)	20 (6, 38)	14 (3, 26)	26 (12, 47)	0.03
Penumbra (ml)	64 (32, 89)	57 (32, 79)	65 (43, 102)	0.40
Final infarct (ml)	13.9 (3.8, 61.4)	8.7 (1.7, 22.4)	24.5 (6.5, 139.1)	**0.01**
Outcome				
mRS score	2 (1, 4)	1 (1, 2)	5 (4, 5)	**<0.001**
Mortality	4 (6.7%)	0 (0%)	4 (14.3%)	**0.04**
Any ICH	20 (33.3%)	9 (28.1%)	11 (39.3%)	0.36
Hemorrhagic infarct	14 (23.3%)	7 (21.9%)	7 (25.0%)	0.78
Parenchymal hemorrhage	6 (10.0%)	2 (6.3%)	4 (14.3%)	0.40

The Mann–Whitney *U* test was used for the continuous variables, whereas the chi-square test was used for the categorical variables. Bold type indicates significant results (*P* < 0.05)

*ASPECTS* Alberta Stroke Program Early CT Score, *ICA* internal carotid artery, *ICH* intracerebral hemorrhage, *mRS* modified Rankin Scale, *mTICI* modified Thrombolysis in Cerebral Infarction, *NIHSS* National Institute of Health Stroke Scale, *tPA* tissue-type plasminogen activator

## Results

### Baseline characteristics of the patients with stroke and controls

During the study period, 88 patients with stroke received EVT at the study hospital. After the exclusion of 28 patients because of unavailable or missing blood samples or because informed consent was not provided, 60 patients with fully available blood samples obtained at the three time points remained for analysis. Among those 60 individuals, the most common types of occlusions were ICA and M1 occlusion (73.3%), and successful recanalization was achieved in 71.7% of them (Table 1). Follow-up imaging revealed that 20 patients (33.3%) had hemorrhagic transformation (14 and 6 with HI and PH, respectively). At 90 days, 53.3% of patients achieved functional independence (mRS score 0–2). The patients who achieved functional independence were significantly younger, were predominantly men, more frequently had atrial fibrillation and successful recanalization, and had a smaller initial infarct core volume (Table 1) compared with those who did not achieve functional independence.

**Fig. 1:**
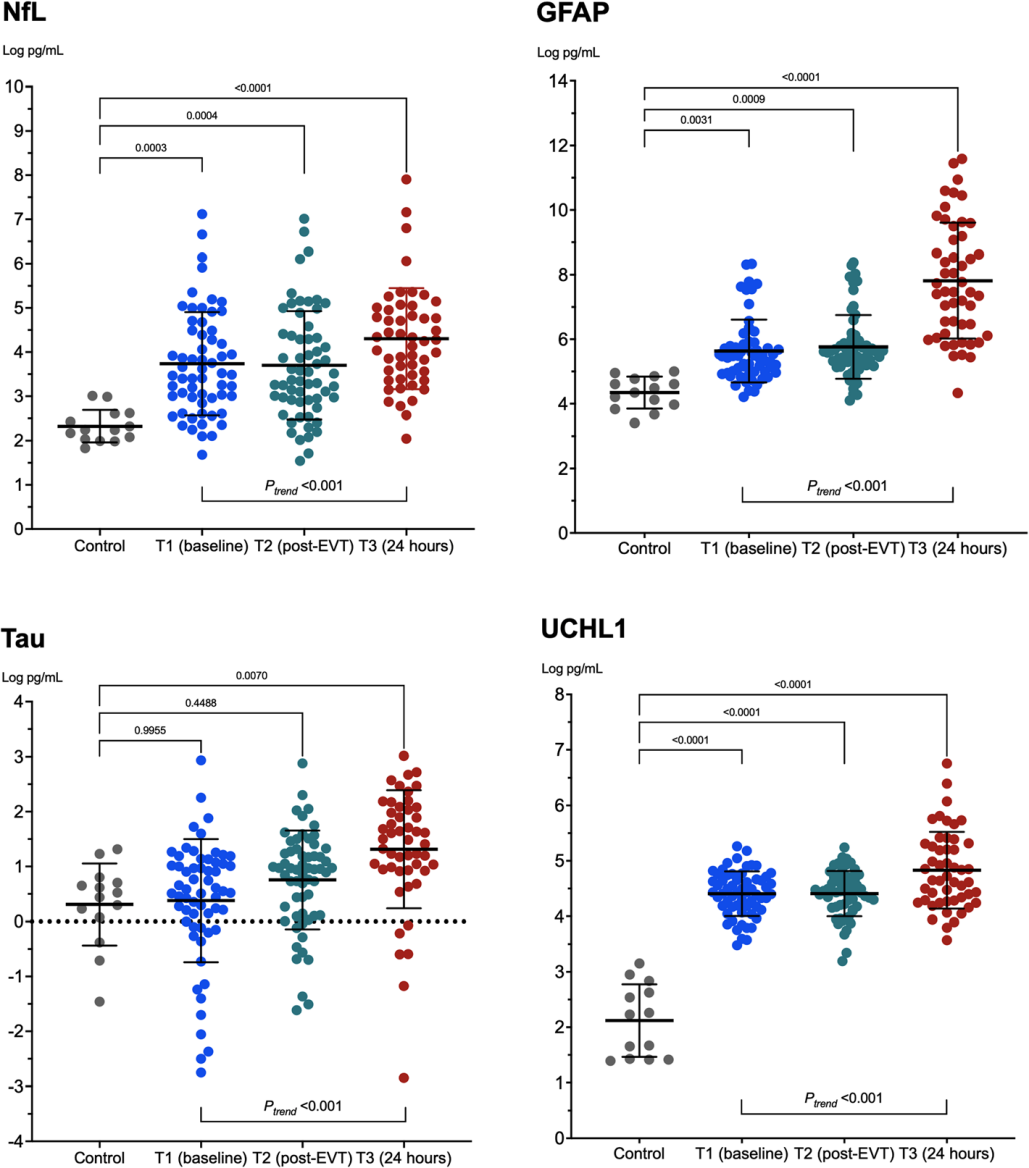
Comparison of biomarkers levels between patients and controls

The mean time between stroke onset and blood draws was 5.13 ± 4.47 h for T1, 5.54 ± 4.66 h for T2, and 27.21 ± 6.23 h for T3. In addition, 14 participants without stroke were included as controls. A comparison of baseline characteristics and biomarker levels between the patients and controls is presented in Table 2. The natural log–transformed plasma levels of NfL, GFAP, tau, and UCHL1 in the controls were 2.33 ± 0.37, 4.35 ± 0.50, 0.31 ± 0.75, and 1.95 ± 0.91, respectively, whereas those of the patients with stroke at T1 (before EVT) were 3.74 ± 1.17 (*P* < 0.001), 5.63 ± 0.97 (*P* < 0.001), 0.38 ± 1.12 (*P* = 0.82), and 4.41 ± 0.4 (*P* < 0.001; Fig. 1), respectively. Although the controls had a lower mean age than did the patients with stroke (66.0 ± 1.41 years vs 71.2 ± 11.8 years; *P* = 0.001), an age-adjusted comparison yielded similar results (*P* < 0.001, *P* < 0.001, *P* = 0.61, and *P* < 0.001 respectively; Table 2).

**Table 2:** Comparison between the controls and the patients with stroke (at baseline before thrombectomy)

	Control (*n* = 14)	Stroke (*n* = 60)	*P* value
Age	66.0±1.41	71.2±11.8	0.001
Male sex	8 (57.1%)	34 (56.7%)	0.97
Hypertension	12 (85.7)	39 (65.0%)	0.20
Diabetes mellitus	5 (35.7%)	22 (36.7%)	0.95
Hyperlipidemia	10 (71.4%)	33 (55.0%)	0.37
Atrial fibrillation	5 (35.7%)	37 (61.7%)	0.13
NfL pg/ml	9.44 (7.73, 13.0)	33.3 (19.6, 88.9)	0.046
GFAP pg/mL	82.9 (55.3, 114.0)	238.8 (146.0, 330.5)	0.015
Tau pg/mL	1.62 (1.12, 2.00)	1.71 (0.97, 2.87)	0.26
UCHL1 pg/mL	7.30 (4.13, 13.5)	83.6 (64.8, 107.6)	<0.001
Log NfL	2.33±0.37	3.74±1.17	<0.001
Log GFAP	4.35±0.50	5.63±0.97	<0.001
Log Tau	0.31±0.75	0.38±1.12	0.61
Log UCHL1	1.95±0.91	4.41±0.40	<0.001

The count and continuous variables are expressed as numbers (percentages) and as mean ± standard deviations, respectively, except for the raw biomarker data, which are presented as medians (first quartile, third quartile) because of their nonnormal distributions. The Mann–Whitney *U* test was used for the raw biomarker data, a *t* test was used for the natural log–transformed biomarkers, and the *P* values for biomarkers were adjusted for age

### Time trends of biomarkers in patients with stroke

**Fig. 2:**
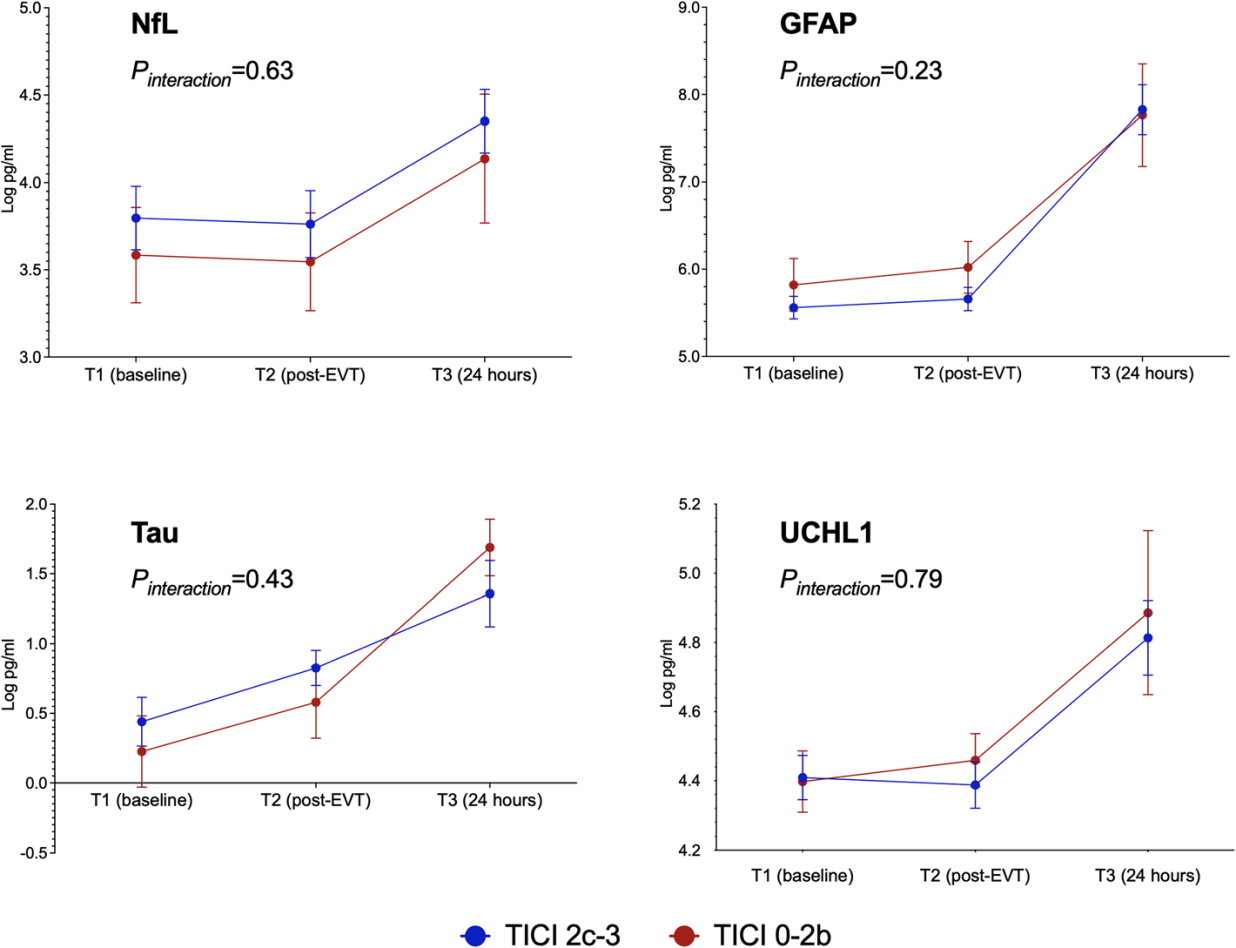
Changes in biomarkers levels in patients with successful recanalization (modified Thrombolysis in Cerebral Infarction [mTICI] score 2c or 3) versus unsuccessful recanalization (mTICI score 0–2b)

**Fig. 3:**
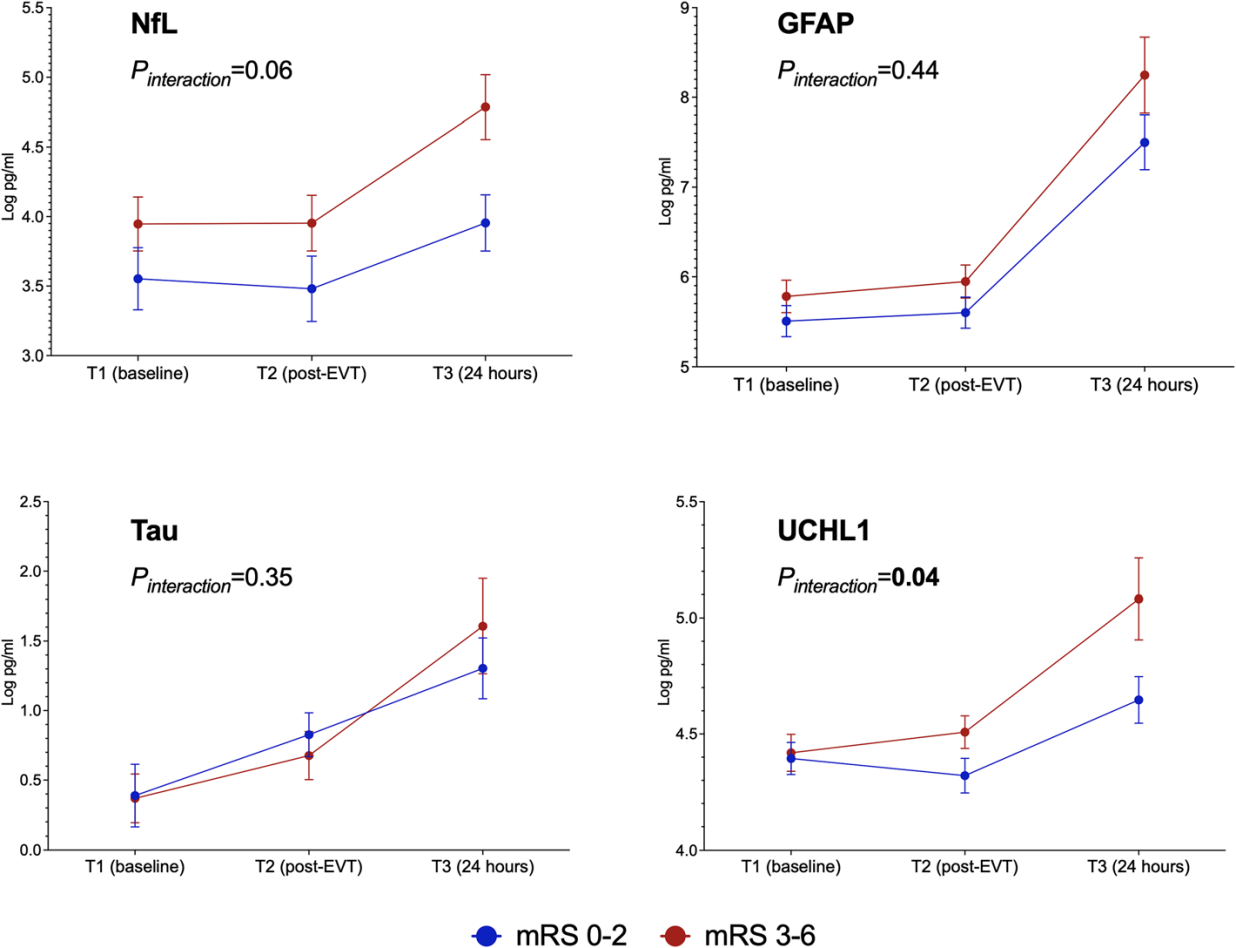
Changes in biomarkers levels in patients with death or disability (modified Rankin Scale [mRS] score 3–6) versus functional independency (mRS score 0–2) at 90 days after stroke

**Fig. 4:**
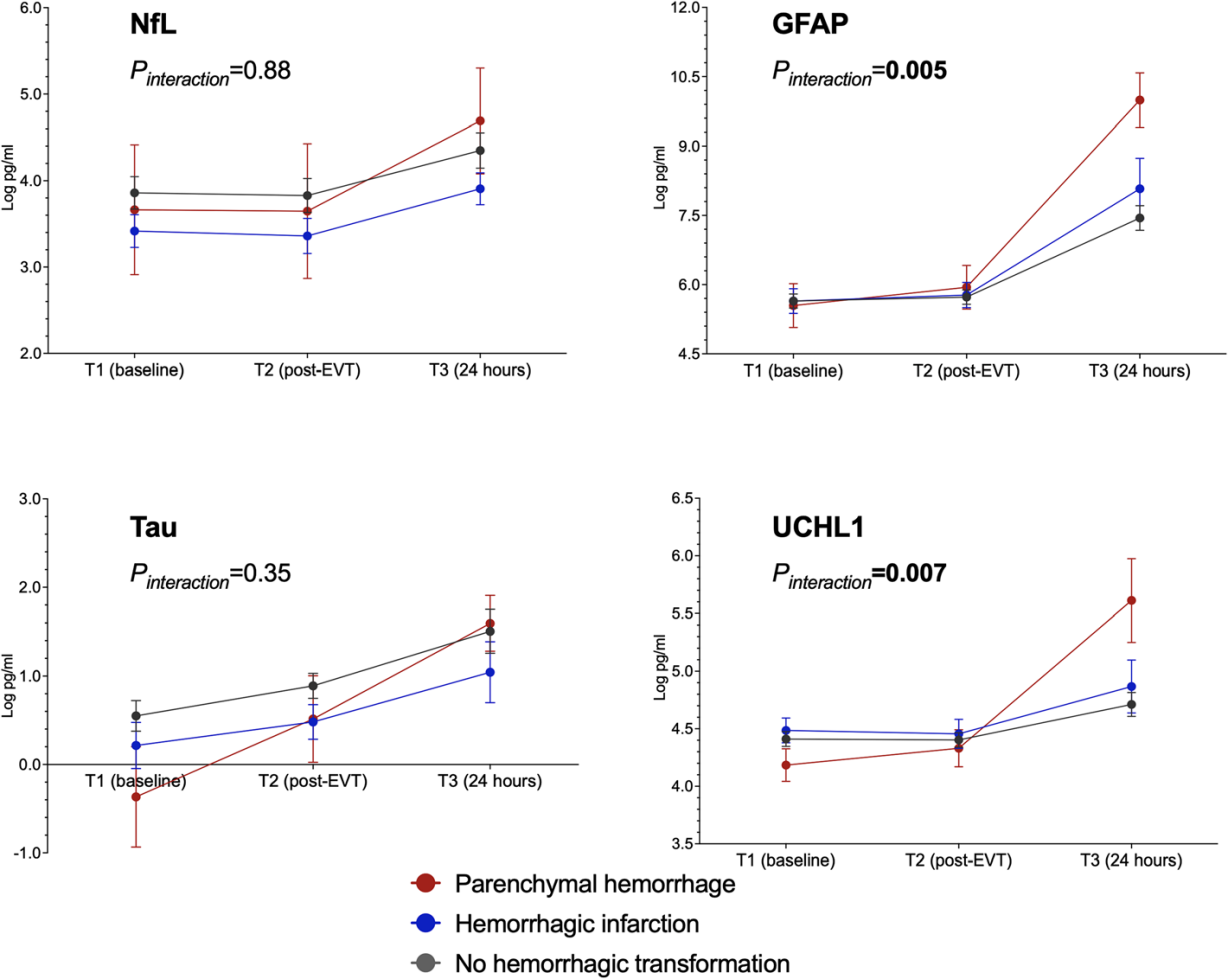
Comparison of changes in biomarker levels between patients stratified according to type of hemorrhagic transformation (no hemorrhage, hemorrhagic infarction, or parenchymal hemorrhage).

Figure 1 presents the serial changes in the levels of each biomarker in the patients with stroke. The levels of all four biomarkers increased significantly with time (*P*_*trend*_ < 0.001, Table 3). The NfL, GFAL, and UCHL1 levels were comparable between T1 and T2 and significantly higher at T3. The temporal change in the levels of all of these plasma biomarkers did not differ significantly between patients with successful recanalization (mTICI grade 2c or 3) and those without (mTICI grade 0–2b; all *P*_*interaction*_ > 0.05; Fig. 2). UCHL1 levels increased more notably in patients with death or disability at 90 days (mRS scores 3–6) than in patients with functional independence (mRS score 0–2; *P*_*interaction*_ = 0.04), and NfL levels exhibited a similar trend between these two groups (*P*_*interaction*_ = 0.06; Fig. 3). When stratified according to the type of hemorrhagic transformation, patients with PH had a significantly higher rate of increase in GFAP (*P*_*interaction*_ = 0.005) and UCHL1 (*P*_*interaction*_ = 0.007) levels compared with that of patients with HI or no ICH, especially at T3 (Fig. 4).

**Table 3:** Changes in biomarker levels according to different outcomes

	Time points	Overall (*n* = 60)	TICI 2c–3 (*n* = 43)	TICI 0–2b (*n* = 17)	mRS 0–2 (*n* = 32)	mRS 3–6 (*n* = 28)	No ICH (*n* = 40)	HI (*n* = 14)	PH (*n* = 6)
NfL	T1	3.74±1.17	3.80±1.19	3.58±1.13	3.55±1.27	3.95±1.03	3.86±1.19	3.42±0.71	3.66±1.84
	T2	3.70±1.23	3.76±1.26	3.55±1.16	3.48±1.33	3.95±1.62	3.83±1.25	3.36±0.76	3.65±1.91
	T3	4.30±1.15	4.35±1.13	4.14±1.22	3.95±1.09	4.79±1.06	4.35±1.22	3.91±0.55	4.69±1.37
		*P*<0.001	*P*_*TICI*_=0.42, *P*_*interaction*_=0.63		*P*_*mRS*_=0.09, *P*_*interaction*_=0.06		*P*_*ICH*_=0.43, *P*_*interaction*_=0.88		
GFAP	T1	5.63±0.97	5.56±0.85	5.82±1.25	5.50±0.99	5.78±0.96	5.64±0.96	5.64±1.00	5.55±1.16
	T2	5.76±0.99	5.66±0.87	6.02±1.23	5.60±0.98	5.95±0.98	5.73±0.98	5.77±1.02	5.94±1.16
	T3	7.81±1.79	7.83±1.77	7.76±1.95	7.50±1.64	8.25±1.94	7.44±1.60	8.08±1.98	9.99±1.32
		*P*<0.001	*P*_*TICI*_=0.41, *P*_*interaction*_=0.23		*P*_*mRS*_=0.09, *P*_*interaction*_=0.44		*P*_*ICH*_=0.14, *P*_*interaction*_=**0.005**		
Tau	T1	0.38±1.12	0.44±1.15	0.23±1.06	0.39±1.28	0.37±0.93	0.55±1.10	0.21±0.98	−0.37±1.39
	T2	0.76±0.90	0.83±0.83	0.58±1.07	0.83±0.89	0.68±0.92	0.89±0.90	0.48±0.73	0.51±1.20
	T3	1.43±1.35	1.36±1.48	1.69±0.67	1.30±1.18	1.61±1.57	1.51±1.48	1.04±1.03	1.60±0.70
		*P*<0.001	*P*_*TICI*_=0.86, *P*_*interaction*_=0.43		*P*_*mRS*_=0.83, *P*_*interaction*_=0.35		*P*_*ICH*_=0.31, *P*_*interaction*_=0.39		
UCHL1	T1	4.41±0.40	4.41±0.42	4.40±0.37	4.39±0.39	4.42±0.42	4.41±0.41	4.49±0.40	4.18±0.35
	T2	4.41±0.41	4.39±0.44	4.46±0.32	4.32±0.42	4.51±0.37	4.40±0.40	4.46±0.47	4.33±0.39
	T3	4.83±0.69	4.81±0.67	4.89±0.79	4.65±0.54	5.08±0.81	4.71±0.62	4.87±0.69	5.61±0.81
		*P*<0.001	*P*_*TICI*_=0.69, *P*_*interaction*_=0.67		*P*_*mRS*_=**0.04**, *P*_*interaction*_=**0.04**		*P*_*ICH*_=0.52, *P*_*interaction*_=**0.007**		

*P*_*TICI*_, *P*_*mRS*_, *P*_*ICH*_ = overall group difference, *P*_*interaction*_= whether groups diverged differently over time. *ICH* intracerebral hemorrhage, *HI* hemorrhagic infarction, *PH* parenchymal hemorrhage

### Associations between biomarkers and outcomes

In the age- and time-adjusted Spearman correlation analyses (Supplemental Table II), initial ASPECTs were negatively correlated with UCHL1 levels at T1 (Spearman’s *ρ* = −0.33, *P* = 0.02) and T2 (*ρ* = −0.41, *P* = 0.005). Final infarct volumes were positively correlated with GFAP levels at T3 (*ρ* = 0.60, *P* < 0.001) and UCHL1 levels at T3 (*ρ* = 0.44, *P* = 0.006) but negatively correlated with tau levels at T1 (*ρ* = −0.35, *P* = 0.02) and T2 (*ρ* = −0.31, *P* = 0.04).

Table 4 presents the associations of biomarkers at different time points with prespecified outcomes. Higher NfL levels at T1 (odds ratio [OR], 2.05 for each natural log–transformed unit increase; 95% confidence interval [CI], 1.03–4.08; *P* = 0.042), T2 (OR, 2.05; 95% CI, 1.05–4.01; *P* = 0.035), and T3 (OR, 3.94; 95% CI, 1.44–10.79; *P* = 0.008) were significant predictors of death or disability at 90 days after adjustment for age, sex, NIHSS score, history of atrial fibrillation, and recanalization status. The level of significance was still reached after false discovery rate adjustment for multiple comparisons. These associations were also detected in the generalized linear mixed model, which accounted for repeated measurements of NfL across the three time points (OR, 2.22; 95% CI, 1.27–3.88; *P* = 0.006). GFAP, tau and UCHL1 levels were not associated with death or disability at 90 days.

**Table 4:** Associations between biomarker levels and clinical outcomes

	Time points	Death or disability (*n*=28)	Any hemorrhagic transformation (*n*=20)	Parenchymal hemorrhage (*n*=6)
		OR (95% CI)	OR (95% CI)	OR (95% CI)
NfL	T1	2.05 (1.03–4.08)*	0.64 (0.35–1.17)	0.94 (0.45–1.98)
	T2	2.05 (1.05–4.01)*	0.65 (0.37–1.16)	0.96 (0.48–1.94)
	T3	3.94 (1.44–10.79)*	0.74 (0.39–1.42)	1.35 (0.64–2.84)
	All	**2.22 (1.27–3.88)**	0.67 (0.40–1.15)	1.05 (0.44–2.55)
GFAP	T1	1.42 (0.73–2.78)	1.01 (0.53–1.91)	0.90 (0.36–2.26)
	T2	1.49 (0.75–2.97)	1.19 (0.64–2.24)	1.21 (0.55–2.70)
	T3	1.28 (0.84–1.95)	**2.39 (1.21**–4.72)*	**2.51 (1.18**–**5.34)**
	All	1.18 (0.91–1.52)	1.21 (0.96–1.53)	1.29 (0.97–1.70)
Tau	T1	1.19 (0.66–2.14)	**0.48 (0.25**–**0.93)**	0.57 (0.29–1.11)
	T2	1.13 (0.50–2.55)	**0.43 (0.20**–**0.95)**	0.73 (0.30–1.78)
	T3	1.14 (0.70–1.86)	0.85 (0.53–1.39)	1.10 (0.57–2.14)
	All	1.11 (0.76–1.63)	0.63 (0.38–1.07)	0.79 (0.46–1.36)
UCHL1	T1	0.83 (0.19–3.70)	0.84 (0.16–4.49)	0.22 (0.03–1.85)
	T2	2.60 (0.53–12.64)	1.04 (0.20–5.35)	0.60 (0.08–4.46)
	T3	2.73 (0.88–8.51)	2.64 (0.83–8.41)	**5.98 (1.31**–**27.24)**
	All	1.76 (0.59–5.23)	1.38 (0.53–3.61)	1.56 (0.56–4.31)

Covariates adjusted for in the logistic regression models were age, sex, NIHSS score, atrial fibrillation, and successful recanalization for death or disability; age, sex, hypertension, and NIHSS score for any hemorrhagic transformation; and none for parenchymal hemorrhage. Generalized linear mixed models were employed to model all of the time points together. Bold type indicates significant results (*P* < 0.05). *False discovery rate–adjusted *P* < 0.05

Higher GFAP levels at T3 were independently associated with the presence of any hemorrhagic transformation (OR, 2.39; 95% CI, 1.21–4.72; *P* = 0.012), and predicted PH (OR, 2.51; 95% CI, 1.18–5.34; *P* = 0.017). Similarly, higher UCHL1 levels at T3 were associated with PH (OR, 5.98; 95% CI, 1.31–27.24; *P* = 0.021). Moreover, higher tau levels at T1 and T2 were inversely associated with any hemorrhagic transformation. However, none of these associations reached statistical significance after the application of the false discovery rate or after their input into the generalized linear mixed model accounting for repeated measurements, excepting the association of higher GFAP levels at T3 with hemorrhagic transformation (Table 4).

## Discussion

The results suggest that in patients with AIS and LVO who underwent EVT, temporal changes in plasma biomarkers levels were associated with neuroimaging and clinical outcomes. The main findings were as follows: (1) higher NfL levels before, immediately after EVT, and 24 h after EVT were associated with unfavorable outcomes; (2) GFAP and UCHL1 levels changed notably within 24 h; and (3) higher GFAP and UCHL1 levels at 24 h after EVT were associated with the severity of hemorrhagic transformation. These findings provide strong evidence of the usefulness of this plasma biomarker panel as a prognostic measure in patients with AIS and LVO undergoing EVT.

Neurofilaments are cytoskeletal proteins expressed exclusively in neurons. When the axon is injured, neurofilaments are released into the extracellular space and then into the cerebrospinal fluid and blood. Thus, they act as a specific biomarker of neuronal injury. They have diagnostic and prognostic values for numerous neurological disorders, such as multiple sclerosis, dementia, traumatic brain injury, and stroke [24]. Studies have reported that plasma NfL levels are elevated soon after cerebral infarction and are related to functional outcomes [11, 25]. In our study, plasma NfL levels were higher among the patients with stroke than among the controls, thus aiding the differentiation of patients with acute neurological disorders from those without. Moreover, NfL levels measured as early as at the time of stroke onset could predict functional outcomes, even before any intervention had been implemented (OR at T1 = 2.07). Relevant studies have also indicated that NfL levels are temporally dynamic: they are elevated in the first few days after acute stroke, peak at day 7, and then remained elevated for 3 months [11, 24, 26]. Consistent with these reports, our findings indicated that the NfL levels increased with time; we further discovered that these increases were more substantial in patients with unfavorable outcomes (*P*_*interaction*_ = 0.06).

GFAP is a brain-specific astrocytic intermediate filament protein that can be released into the cerebrospinal fluid or the blood following brain injury. GFAP levels can thus reflect the severity of neuronal damage and are believed to be higher in patients with ICH than in controls or even in patients with AIS [14, 15, 17]. However, UCHL1, a neuronal cytoplasmic deubiquitinating enzyme abundantly expressed in neurons, is associated with synaptic plasticity and self-repair mechanisms after injury. When GFAP and UCHL1 levels were simultaneously assessed in patients with acute stroke, GFAP levels were usually more useful than were UCHL levels in early differentiation between patients with ICH and those with AIS [17, 27]. However, in our study, which focused on EVT-treated patients, GFAP and UCHL1 levels exhibited comparable temporal changes and clinical associations. For example, the levels of both biomarkers at 24 h after EVT were positively correlated with final infarct volumes and could predict PH (OR, 2.51–5.98). Furthermore, both increased significantly over time in patients with a more severe hemorrhagic transformation. Compared with the GFAP levels, UCHL1 levels were notably higher in the patients with unfavorable outcomes. Overall, these results accord with the previous observation of a close correlation between GFAP level and hemorrhagic stroke, even in patients with AIS who received EVT. They also provide new insights on the comparable relationship identified for UCHL1 level.

Our data indicated that NfL levels are associated with functional dependency but not hemorrhagic transformation, whereas GFAP and UCHL1 levels can predict hemorrhagic transformation but not functional outcomes. Symptomatic ICH after EVT is associated with unfavorable outcomes and higher mortality [28]. However, in our study, the incidence of symptomatic ICH was low (only one case, 1.7%), thus preventing the acquisition of valid statistical results. However, according to the latest Heidelberg Bleeding Classification, PH is closely related to symptomatic ICH and can serve as a surrogate neuroimaging outcome [29]. A larger infarct size could increase the probability of PH; this idea is supported by our study results, which revealed that GFAP and UCHL1 levels not only predicted PH but were also strongly correlated with final infarct volumes. In addition, the rate of increase in UCHL1 level over time was also higher among patients who had unfavorable outcomes (Fig. 2 and Table 2). However, although NfL level was not associated with signs of hemorrhage, hemorrhagic transformation itself did not necessarily correspond to unfavorable outcomes because it may have been caused by recanalization or reperfusion in some cases. Factors that typically predict functional outcomes after EVT include age, initial NIHSS score, prestroke mRS score, ASPECTS, time from onset-to-groin puncture, and glucose level [30]. In our study, NfL levels were not correlated with age (*ρ* = −0.03, *P* = 0.81) or NIHSS score (*ρ* = 0.05, *P* = 0.72), but they independently predicted death or disability in the multivariable-adjusted models. Therefore, the prognostic role of NfL level is based on its direct association with neuronal injury in the event of acute stroke and may even reflect underlying CNS degeneration, which is believed to be linked to unfavorable functional outcomes after stroke.

The four investigated biomarkers exhibited similar patterns in terms of temporal changes: the levels were almost the same at T1 and T2 but were considerably higher at T3. A notable finding is that even within minutes to hours after stroke onset, these plasma biomarkers already reflected neuronal injury caused by the ischemic cascade [31]. However, the prognostic role of plasma biomarkers levels at T2, which we initially hypothesized to be dependent on recanalization status, may not differ substantially from the prognostic role of these levels at T1 because the median puncture-to-recanalization time was only 16 min in our patient group. Moreover, the death of neurons and astrocytes following the ischemic cascade may continue for several hours and even days after successful recanalization. This explains why the plasma biomarkers levels peaked at T3 and were unaffected by recanalization status.

Notably, a similar study reported that plasma levels of NfL, tau, and GFAP also increased over time in patients treated with EVT and were correlated with stroke clinical severity and outcomes [26]. They reported that plasma tau and GFAP levels peaked at 24–72 h and were lower at 3 months after stroke. However, plasma NfL levels continued to increase even at 3 months, possibly reflecting the chronic post-ischemic Wallerian degeneration of myelinated axons. Our study results echo their findings regarding the initial increase in plasma biomarker levels after stroke and their potential prognostic roles.

Our study has several strengths. The included patients were recruited from a prospective stroke registry and had relatively complete clinical and neuroimaging profiles. Blood samples were measured thrice, not only once, thus providing a clear view of the temporal relationship between plasma biomarker levels and clinical outcomes. Also, we used a modern multiplex single-molecule array to simultaneously quantify several relevant biomarkers. Our study also has several limitations. First, the sample size was relatively small, limiting the statistical power of our findings. Nevertheless, the biomarkers exhibited clear associations with clinical and neuroimaging outcomes. Our study can serve as a pilot study, and our results can be verified in larger-scale investigations. Second, even in a prospective registration setting, some factors for which adjustments were necessary in the multivariable analysis may not have been adjusted. Third, we did not determine a clear cutoff value for the biomarkers for predicting outcomes, unlike several relevant studies [10, 13, 17]. However, variations among stroke centers in terms of patient profiles, acute treatment strategies, and standard care protocols may complicate the acquisition of a universally applicable set of values for predicting outcomes. Finally, the plasma biomarkers levels before stroke onset were unknown. A history of neurological diseases prior to AIS may have affected the patients’ biomarker levels. Nevertheless, the results of comparisons between patients with stroke and controls and those for temporal changes in plasma biomarker levels indicate that this limitation likely did not have any major effects on the findings.

In conclusions, in patients with AIS and LVO who underwent EVT, higher plasma NfL levels were predictive of unfavorable functional outcomes. Substantial increase in UCHL1 levels after stroke—measured before, immediately after, and 24 h after EVT—was observed in the patients with unfavorable outcomes; moreover, temporal change in of GFAP and UCHL1 levels were associated with more severe types of hemorrhagic transformation, especially PH. Our findings jointly imply that these biomarkers have clinical value for prognostic prediction. Larger-scale studies are warranted to verify our findings.

## Supplementary Information

**Additional file 1.** Supplemental Tables I and II.

## Data Availability

The datasets used and/or analyzed during the current study are available from the corresponding author on reasonable request.

## Abbreviations

AIS: Acute ischemic stroke
ASPECTS: Alberta Stroke Program Early CT Score
EVT: Endovascular thrombectomy
GFAP: Glial fibrillary astrocytic protein
HI: Hemorrhagic infarct
ICH: Intracerebral hemorrhage
LVO: Large vessel occlusion
NfL: Neurofilament light chain
NIHSS: National Institute of Health Stroke Scale
PH: Parenchymal hemorrhage
UCHL1: Ubiquitin C-terminal hydrolase L1

## References

[1] Smith WS, Lev MH, English JD, Camargo EC, Chou M, Johnston SC, Gonzalez G, Schaefer PW, Dillon WP, Koroshetz WJ, Furie KL (2009). Significance of large vessel intracranial occlusion causing acute ischemic stroke and TIA. Stroke..

[2] Goyal M, Menon BK, van Zwam WH, Dippel DW, Mitchell PJ, Demchuk AM (2016). Endovascular thrombectomy after large-vessel ischaemic stroke: a meta-analysis of individual patient data from five randomised trials. Lancet (London, England).

[3] Mizuma A, You JS, Yenari MA (2018). Targeting reperfusion injury in the age of mechanical thrombectomy. Stroke..

[4] Stoll G, Nieswandt B (2019). Thrombo-inflammation in acute ischaemic stroke - implications for treatment. Nat Rev Neurol..

[5] Tang SC, Arumugam TV, Xu X, Cheng A, Mughal MR, Jo DG, Lathia JD, Siler DA, Chigurupati S, Ouyang X, Magnus T, Camandola S, Mattson MP (2007). Pivotal role for neuronal Toll-like receptors in ischemic brain injury and functional deficits. Proc Natl Acad Sci U S A..

[6] Esenwa CC, Elkind MS (2016). Inflammatory risk factors, biomarkers and associated therapy in ischaemic stroke. Nat Rev Neurol..

[7] Zhong C, Yang J, Xu T, Xu T, Peng Y, Wang A, Wang J, Peng H, Li Q, Ju Z, Geng D, Zhang Y, He J, For the CATIS Investigators (2017). Serum matrix metalloproteinase-9 levels and prognosis of acute ischemic stroke. Neurology..

[8] Montellano FA, Ungethum K, Ramiro L, Nacu A, Hellwig S, Fluri F, et al. Role of blood-based biomarkers in ischemic stroke prognosis: a systematic review. Stroke. 2021;52(2):543-551, 2, DOI: 10.1161/STROKEAHA.120.029232. 10.1161/STROKEAHA.120.029232 33430636

[9] Heller C, Foiani MS, Moore K, Convery R, Bocchetta M, Neason M, Cash DM, Thomas D, Greaves CV, Woollacott IOC, Shafei R, van Swieten JC, Moreno F, Sanchez-Valle R, Borroni B, Laforce Jr R, Masellis M, Tartaglia MC, Graff C, Galimberti D, Rowe JB, Finger E, Synofzik M, Vandenberghe R, de Mendonca A, Tagliavini F, Santana I, Ducharme S, Butler CR, Gerhard A, Levin J, Danek A, Frisoni G, Sorbi S, Otto M, Heslegrave AJ, Zetterberg H, Rohrer JD (2020). Plasma glial fibrillary acidic protein is raised in progranulin-associated frontotemporal dementia. J Neurol Neurosurg Psychiatry..

[10] Chen CH, Cheng YW, Chen YF, Tang SC, Jeng JS (2020). Plasma neurofilament light chain and glial fibrillary acidic protein predict stroke in CADASIL. J Neuroinflammation..

[11] Khalil M, Teunissen CE, Otto M, Piehl F, Sormani MP, Gattringer T, Barro C, Kappos L, Comabella M, Fazekas F, Petzold A, Blennow K, Zetterberg H, Kuhle J (2018). Neurofilaments as biomarkers in neurological disorders. Nat Rev Neurol.

[12] Lambertsen KL, Soares CB, Gaist D, Nielsen HH (2020). Neurofilaments: The C-Reactive Protein of Neurology. Brain Sci..

[13] Katsanos AH, Makris K, Stefani D, Koniari K, Gialouri E, Lelekis M, Chondrogianni M, Zompola C, Dardiotis E, Rizos I, Parissis J, Boutati E, Voumvourakis K, Tsivgoulis G (2017). Plasma glial fibrillary acidic protein in the differential diagnosis of intracerebral hemorrhage. Stroke..

[14] Perry LA, Lucarelli T, Penny-Dimri JC, McInnes MD, Mondello S, Bustamante A (2019). Glial fibrillary acidic protein for the early diagnosis of intracerebral hemorrhage: Systematic review and meta-analysis of diagnostic test accuracy. Int J Stroke.

[15] Zhang J, Zhang CH, Lin XL, Zhang Q, Wang J, Shi SL (2013). Serum glial fibrillary acidic protein as a biomarker for differentiating intracerebral hemorrhage and ischemic stroke in patients with symptoms of acute stroke: a systematic review and meta-analysis. Neurol Sci..

[16] Montellano FA, Ungethüm K, Ramiro L, Nacu A, Hellwig S, Fluri F, et al. Role of blood-based biomarkers in ischemic stroke prognosis: a systematic review. Stroke. 2021;52(2):543–51. 10.1161/STROKEAHA.120.029232. 10.1161/STROKEAHA.120.029232 33430636

[17] Ren C, Kobeissy F, Alawieh A, Li N, Li N, Zibara K, Zoltewicz S, Guingab-Cagmat J, Larner SF, Ding Y, Hayes RL, Ji X, Mondello S (2016). Assessment of serum UCH-L1 and GFAP in acute stroke patients. Sci Rep..

[18] Adams HP, Bendixen BH, Kappelle LJ, Biller J, Love BB, Gordon DL, Marsh EE (1993). Classification of subtype of acute ischemic stroke. Definitions for use in a multicenter clinical trial. TOAST. Trial of Org 10172 in Acute Stroke Treatment. Stroke..

[19] Powers WJ, Rabinstein AA, Ackerson T, Adeoye OM, Bambakidis NC, Becker K, Biller J, Brown M, Demaerschalk BM, Hoh B, Jauch EC, Kidwell CS, Leslie-Mazwi TM, Ovbiagele B, Scott PA, Sheth KN, Southerland AM, Summers DV, Tirschwell DL (2019). Guidelines for the Early Management of Patients With Acute Ischemic Stroke: 2019 Update to the 2018 Guidelines for the Early Management of Acute Ischemic Stroke: A Guideline for Healthcare Professionals From the American Heart Association/American Stroke Association. Stroke..

[20] Chen CH, Tang SC, Tsai LK, Hsieh MJ, Yeh SJ, Huang KY, Jeng JS (2014). Stroke code improves intravenous thrombolysis administration in acute ischemic stroke. PLoS One..

[21] Chu HJ, Tang SC, Lee CW, Jeng JS, Liu HM (2018). Endovascular thrombectomy for acute ischemic stroke: a single-center experience in Taiwan. J Formos Med Assoc..

[22] Goyal M, Fargen KM, Turk AS, Mocco J, Liebeskind DS, Frei D, Demchuk AM (2014). 2C or not 2C: defining an improved revascularization grading scale and the need for standardization of angiography outcomes in stroke trials. J Neurointerv Surg..

[23] von Kummer R, Broderick JP, Campbell BC, Demchuk A, Goyal M, Hill MD (2015). The Heidelberg Bleeding Classification: classification of bleeding events after ischemic stroke and reperfusion therapy. Stroke..

[24] Tiedt S, Duering M, Barro C, Kaya AG, Boeck J, Bode FJ, Klein M, Dorn F, Gesierich B, Kellert L, Ertl-Wagner B, Goertler MW, Petzold GC, Kuhle J, Wollenweber FA, Peters N, Dichgans M (2018). Serum neurofilament light: A biomarker of neuroaxonal injury after ischemic stroke. Neurology..

[25] Uphaus T, Bittner S, Groschel S, Steffen F, Muthuraman M, Wasser K (2019). NfL (Neurofilament Light Chain) Levels as a predictive marker for long-term outcome after ischemic stroke. Stroke..

[26] Pujol-Calderon F, Zetterberg H, Portelius E, Lowhagen Henden P, Rentzos A, Karlsson JE, et al. Prediction of outcome after endovascular embolectomy in anterior circulation stroke using biomarkers. Transl Stroke Res. 2021. 10.1007/s12975-021-00905-5. 10.1007/s12975-021-00905-5 PMC8766380 33723754

[27] Luger S, Jæger HS, Dixon J, Bohmann FO, Schaefer J, Richieri SP (2020). Diagnostic accuracy of glial fibrillary acidic protein and ubiquitin carboxy-terminal hydrolase-L1 serum concentrations for differentiating acute intracerebral hemorrhage from ischemic stroke. Neurocrit Care..

[28] Hao Y, Yang D, Wang H, Zi W, Zhang M, Geng Y, Zhou Z, Wang W, Xu H, Tian X, Lv P, Liu Y, Xiong Y, Liu X, Xu G, Liu C, Shi Z, Zhang J, Lin H, Lin M, Hu Z, Deng X, Wan Y, Zhang J, Shi Z, Qu M, Huang X, Quan T, Guan S, Chen L, Li X, Wang S, Yang S, Liu W, Wei D, Wang Z, Liu X, Guo F, Yang S, Zheng D, Wu X, Zeng Y, Tu M, Jin P, Liu Y, Li H, Fang J, Xiao G, for the ACTUAL Investigators (Endovascular Treatment for Acute Anterior Circulation Ischemic Stroke Registry) (2017). Predictors for symptomatic intracranial hemorrhage after endovascular treatment of acute ischemic stroke. Stroke..

[29] Neuberger U, Mohlenbruch MA, Herweh C, Ulfert C, Bendszus M, Pfaff J (2017). Classification of bleeding events: comparison of ECASS III (European Cooperative Acute Stroke Study) and the new Heidelberg Bleeding Classification. Stroke..

[30] Ben Hassen W, Raynaud N, Bricout N, Boulouis G, Legrand L, Ferrigno M, Kazemi A, Bretzner M, Soize S, Farhat W, Seners P, Turc G, Zuber M, Oppenheim C, Cordonnier C, Naggara O, Henon H (2020). MT-DRAGON score for outcome prediction in acute ischemic stroke treated by mechanical thrombectomy within 8 hours. J Neurointerv Surg..

[31] Xing C, Arai K, Lo EH, Hommel M (2012). Pathophysiologic cascades in ischemic stroke. Int J Stroke..

