# Supplementary material for: Plasma neurofilament light chain level predicts outcomes in stroke patients receiving endovascular thrombectomy
Source: J Neuroinflammation. 2021 Sep 12;18:195. doi: 10.1186/s12974-021-02254-4 (PMC8436486; doi:10.1186/s12974-021-02254-4)
Supplement: Supplementary file 1 — Additional file 1. Supplemental Tables I and II. [file 12974_2021_2254_MOESM1_ESM.docx]

**Plasma Neurofilament Light Chain Level Predicts Outcomes in Stroke Patients Receiving Endovascular Thrombectomy**

**Supplemental Materials**

Supplemental Table I – II

**Supplemental Table I. Comparison between patients with and without hemorrhagic transformation.**

|  | no hemorrhagic transformation (n=40) | any hemorrhagic transformation (n=20) | *P* |
| --- | --- | --- | --- |
| Age | 70.5±12.2 | 72.7±11.0 | 0.43 |
| Male sex | 24 (60.0%) | 10 (50.0%) | 0.46 |
| Hypertension | 21 (52.5%) | 18 (90.0%) | **0.004** |
| DM | 12 (30.0%) | 10 (50.0%) | 0.13 |
| Hyperlipidemia | 23 (57.5%) | 10 (50.0%) | 0.58 |
| Atrial fibrillation | 23 (57.5%) | 14 (70.0%) | 0.35 |
| tPA administration | 11 (27.5%) | 5 (25.0%) | 0.84 |
| NIHSS | 15.1±6.9 | 18.8±6.5 | 0.09 |
| Stroke subtype |  |  | 0.92 |
| CE | 28 (70.0%) | 15 (75.0%) |  |
| LAA | 7 (17.5%) | 3 (15.0%) |  |
| Others | 5 (12.5%) | 2 (10.0%) |  |
| ICA/M1 occlusion | 28 (70.0%) | 16 (80.0%) | 0.41 |
| EVT procedure |  |  |  |
| Onset-to-puncture | 216 (118, 389) | 210 (146, 322) | 0.85 |
| Onset-to-recanalized | 234 (138, 405) | 234 (165, 385) | 0.78 |
| mTICI 2c-3 | 30 (75.0%) | 13 (65.0%) | 0.42 |
| Neuroimaging |  |  |  |
| ASPECTS | 9 (7, 10) | 8 (6, 9) | 0.10 |
| Initial core (ml) | 17 (6, 26) | 33 (4, 58) | 0.15 |
| Penumbra (ml) | 53 (31.5, 86.5) | 67 (43, 105) | 0.29 |
| Final infarct (ml) | 6.9 (1.7, 24.4) | 25.8 (16, 96.6) | **0.001** |
| Outcome |  |  |  |
| mRS | 2 (1, 4) | 3 (2, 5) | 0.18 |
| Mortality | 2 (5.0%) | 2 (10.0%) | 0.59 |
| Baseline biomarkers |  |  |  |
| Log NfL | 3.86±1.19 | 3.49±1.12 | 0.25 |
| Log GFAP | 5.64±0.96 | 5.61±1.02 | 0.68 |
| Log Tau | 0.55±1.10 | 0.04±1.11 | 0.08 |
| Log UCHL1 | 4.41±0.41 | 4.40±0.40 | 0.86 |

Mann–Whitney U test for continuous variables, and chi-square test for categorical variables.

Abbreviation: ASPECTS, Alberta Stroke Program Early CT Score; CE, cardioembolism; ICA, internal carotid artery; ICH, intracerebral hemorrhage; LAA, large-artery atherosclerosis; mTICI, modified Thrombolysis In Cerebral Infarction; NIHSS, National Institute of Health Stroke Scale; tPA, tissue-type plasminogen activator.

Numbers in bold indicated statistical significance (*P* < 0.05).

**Supplemental Table II.** Correlation between biomarkers and neuroimaging parameters.

|  |  | ASPECTS | Initial core | Initial penumbra | Final infarct |
| --- | --- | --- | --- | --- | --- |
| T1 | NfL | -0.06 (0.70) | -0.16 (0.31) | -0.03 (0.83) | -0.01 (0.92) |
|  | GFAP | -0.14 (0.36) | -0.25 (0.09) | -0.10 (0.51) | -0.01 (0.95) |
|  | Tau | 0.02 (0.92) | -0.30 (0.05) | -0.07 (0.65) | **-0.35 (0.02)** |
|  | UCHL1 | **-0.33 (0.02)** | -0.08 (0.60) | -0.03 (0.85) | -0.01 (0.99) |
| T2 | NfL | -0.06 (0.70) | -0.13 (0.41) | -0.02 (0.88) | 0.002 (0.99) |
|  | GFAP | -0.26 (0.09) | -0.21 (0.17) | -0.16 (0.28) | 0.17 (0.25) |
|  | Tau | -0.09 (0.54) | **-0.32 (0.03)** | -0.08 (0.62) | **-0.31 (0.04)** |
|  | UCHL1 | **-0.41 (0.005)** | 0.01 (0.95) | -0.06 (0.70) | 0.13 (0.38) |
| T3 | NfL | -0.03 (0.88) | -0.02 (0.91) | -0.06 (0.74) | 0.27 (0.10) |
|  | GFAP | -0.14 (0.41) | -0.04 (0.81) | -0.09 (0.60) | **0.60 (<0.001)** |
|  | Tau | -0.26 (0.11) | -0.07 (0.68) | -0.04 (0.83) | 0.17 (0.31) |
|  | UCHL1 | -0.27 (0.11) | -0.10 (0.57) | -0.07 (0.68) | **0.44 (0.006)** |

Values are presented as ρ (*P* value) by Spearman’s rank sum test after adjustment for age and onset-to-groin puncture time.
